# Physiological Responses and Transcriptome Analysis of *Camellia reticulata* Under Low-Temperature Stress

**DOI:** 10.3390/genes16050503

**Published:** 2025-04-27

**Authors:** Yawen Wu, Jian Dong, Ran Pu, Pan Wang, Timei Sun, Jie Li, Jingli Zhang, Tian Bai

**Affiliations:** 1 College of Landscape and Horticulture, Yunnan Agricultural University, Kunming 650201, China; yawenwu@ynau.edu.cn (Y.W.); dongjian9722@163.com (J.D.); puran1026@163.com (R.P.); 15808807252@163.com (P.W.); 19988120814@163.com (T.S.); lijie_mas@163.com (J.L.); 2 National Rhododendron Germplasm Resource Bank, Yunnan Agricultural University, Kunming 650201, China

**Keywords:** cold stress, physiological index, transcriptome, transcription factor, *Camellia reticulata*

## Abstract

**Background:** *Camellia* species are highly ornamental but sensitive to habitat temperature, making cross-border domestication challenging. **Methods:** In this study, physiological indicators and transcriptome data of *Camellia reticulata* ‘shizhitou’ were analyzed to identify key factors involved in the response to cold. **Results:** The findings provide a scientific basis for the conservation of *Camellia* germplasm resources and breeding of cold-tolerant varieties. Under prolonged low-temperature stress, significant changes were observed in the physiological indices of *C. reticulata* ‘shizhitou’. Among soluble substances, soluble protein content continuously increased, while soluble sugar content exhibited a fluctuation pattern of increase–decrease–increase. Under prolonged low-temperature stress, significant changes were observed in the physiological indexes of *C. reticulata* ‘shizhitou’, while soluble sugar content exhibited a fluctuation pattern of increase–decrease–increase. Overall, soluble sugar and soluble protein contents were significantly positively correlated. Chlorophyll content initially decreased and then increased, whereas peroxidase (POD) and catalase (CAT) activities fluctuated and were negatively correlated with chlorophyll content. Malondialdehyde (MDA) content showed an irregular fluctuation trend. A total of 56,424 unigenes were obtained by transcriptome sequencing, of which 39,278 were annotated, while 10,816 differentially expressed genes (DEGs) were identified, including 5748 up-regulated and 5068 down-regulated genes, with 143 DEGs commonly shared across conditions. **Congclusions:** Gene Ontology (GO) and Kyoto Encyclopedia of Genes and Genomes (KEGG) analyses revealed that low-temperature stress significantly influenced glucose metabolism, lipid metabolism, and amino acid metabolism, and the core pathways of cold stress included zeatin synthesis, hormone signaling, and galactose metabolism. Both physiological responses and transcriptome-based enrichment of DEGs indicate that the redox system and metabolic pathways play crucial regulatory roles in *C. reticulata* under cold stress.

## 1. Introduction

*Camellia reticulata*, belonging to the genus *Camellia* of the family Theaceae, is also known as Yunnan *Camellia*. It is an evergreen shrub or small tree, primarily distributed in the mountain forests west of Chuxiong City and east of Tengchong City in Yunnan Province, China. As a primitive species of the genus *Camellia*, it possessses ornamental value and research value [1]. With the rapid development of China’s flower industry and advancements in breeding and cultivation techniques, the standardization and commercialization of traditional ornamental plants have progressively improved [2]. As a traditional ornamental plant in China, *C. reticulata* exhibits both ornamental and medicinal properties, and it is widely traded in flower markets, exhibitions, and e-commerce platforms. Additionally, it has gained market presence in Asia, Europe, and North America [3]. However, due to its preference for a warm and humid climate, *C. reticulata* exhibits poor cold and heat tolerance and is highly sensitive to temperature fluctuations, posing challenges for large-scale cultivation and commercial expansion [4]. Currently, the market of *C. reticulata* is primarily localized, and the cultivation base is concentrated in Yunnan native flower industry parks and large growers [5]. Although the domestic and international cultivation of *C. reticulata* remains in its early stages, enterprises and research institutions have initiated relevant studies and trial planting. Therefore, elucidating the cold resistance mechanism of *C. reticulata* is crucial for enhancing its adaptability and promoting its broader commercial application.

Early studies on the cold resistance of *Camellia* plants mainly focused on the analysis of physiological indexes, which provided an important basis for the study of the cold stress of *Camellia*. Under low-temperature stress, the chloroplast membrane structure of *Camellia* was damaged, chlorophyll content decreased [6,7], and photosynthetic efficiency decreased. Additionally, under low-temperature stress, the content of malondialdehyde (MDA) increased significantly, the lipid peroxidation of the cell membrane was enhanced, and the cell membrane was damaged [8]. At low temperatures, the activities of peroxidase (POD) and superoxide dismutase (CAT) in *Camellia* plants increased significantly, and the content of soluble protein increased, while the content of soluble sugar decreased. There was a significant correlation between soluble protein, soluble sugar, POD, and CAT, resulting in great differences in temperature thresholds for different *Camellia* plants and their varieties [9].

Using homologous cloning, studies have shown that the expression levels of *CsCBF1* and *CsICE1* genes in winter-hardy *Camellia* species significantly increase under low-temperature conditions [10]. Moreover, the expression levels of *CsCBF1* and *CsICE1* in three cold-tolerant germplasms were significantly higher than those in three cold-sensitive germplasms [11]. *Camellia* has a rapid and complex mechanism for sensing, converting, and responding to cold stress signals, as low-temperature signals are detected by cell membrane receptors and then converted into Ca^2+^, ROS, ABA, osmotic stress, and other signals [12]. Therefore, the regulation of the transcription level is an important form of regulation in the regulation of plant life activities [13].

High-throughput transcriptome sequencing technology has been widely used in the study of the mechanism of plant responses to biological and abiotic stresses, and genes involved in defense, nutrient transport, signal transduction, and secondary metabolism have been identified, which has played a significant role in revealing the molecular mechanisms of plant resistance [14,15,16,17].

In summary, research on the cold resistance mechanisms of *Camellia* plants at the gene level and physiological level has revealed considerable diversity and complexity. Physiological mechanisms and gene regulation are different, and there are many types. Therefore, further experimental validation is required to confirm the cold resistance traits observed in these varieties and their value for promotion and application and to establish a complete low-temperature stress map and planting resource database of *C. reticulata.* This cultivar is widely cultivated for its ornamental and economic value; however, its cold tolerance mechanisms remain poorly understood. Investigating its cold stress response will contribute to breeding efforts and resource conservation, providing a scientific basis for studying cold tolerance in *Camellia*.

Investigating the cold-tolerance mechanism of *C. reticulata* ‘Shizhitou’ under cold stress is of the utmost importance. ‘Shizhitou’ is extensively used and cultivated among *C. reticulata* varieties. Therefore, in this study, using *C. reticulata* ‘Shizhitou’ as the experimental material, we conducted sustained cold stress treatments, measured physiological indicators, and performed RNA-seq transcriptome analysis. A dedicated transcriptome database was established to identify candidate genes associated with low-temperature stress response. Through differential expression analysis, we screened significantly regulated genes under cold conditions and further explored the involved metabolic pathways and transcription factors related to cold stress adaptation. This provided theoretical support for breeding and cultivating germplasm resources of *Camellia*.

## 2. Materials and Methods

### 2.1. Treatment of Test Materials

Using *C. reticulata* ‘Shizhitou’ perennial plants as material, the artificial climate room temperature was (25 ± 1) °C/(15 ± 1) °C, the air relative humidity was (75 ± 5)%, and the culture was carried out by light intensity (300 μmol·m^−2^·s^−1^) for 15 days. These are its optimal growth conditions. The artificial climate chamber was programmed at a temperature ramp rate of 4 °C/h (cooling rate of 2 °C/h) with consistent light and humidity conditions maintained throughout. Samples were taken at low temperature at 4 °C for 4 h (T1), 12 h (T2), 24 h (T3), 48 h (T4), and 96 h (T5), respectively, and the materials not treated with cooling were used as the control (CK). After the leaf materials were cleaned, they were dried, quickly put in liquid nitrogen, and stored in the refrigerator at −80 °C for later use. Biological replicates were performed 3 times per treatment.

### 2.2. Measurement of Physiological Indexes

The contents of chlorophyll, soluble sugar, soluble protein, malondialdehyde, free proline, and the activities of peroxidase (POD) and catalase (CAT) were determined using the method described in [18].

### 2.3. Transcriptome Sequencing and Data Analysis

Sample extraction, cDNA library construction, transcriptome sequencing, and data processing were carried out in Baimaik, and data analysis was completed using the BMKCloud data analysis platform. DIAMOND was used to compare Unigene sequences with NR, Swiss-Prot, COG, KOG, eggNOG, and KEGG databases. KEGG Orthology results were obtained by KOBAS, and GO annotations were analyzed by InterProScan [19,20,21,22,23,24,25,26,27]. Reads were compared to the Unigene library by Bowtie, the expression quantity was estimated by RSEM, and the expression abundance was expressed by FPKM value [28,29,30]. FDR < 0.01 and FLOG2 (FC)∣ ≥ 2 were set to screen differentially expressed genes for hierarchical cluster analysis, and the differential genes were enriched by the GO function and KEGG pathway to reveal key regulatory genes and metabolic pathways under low-temperature stress [25,26,27].

### 2.4. QRT-PCR Analysis

Ten randomly selected differentially expressed genes (DEGs) annotated to Gene Ontology (GO) terms were analyzed. Total RNA from these genes was reverse-transcribed into cDNA and subjected to quantitative real-time polymerase chain reaction (qRT-PCR) analysis using primer sequences listed in Table 1. The *Actin* gene served as the internal reference, and relative gene expression levels were calculated via the 2^−ΔΔCt^ method. Three independent biological replicates were performed for statistical validation.

## 3. Results

### 3.1. Effect and Analysis of Cold Stress on Physiology of C. reticulata Slices

#### 3.1.1. Change of Soluble Substance Content in Low Temperature Stress Treatment

**Figure 1:**
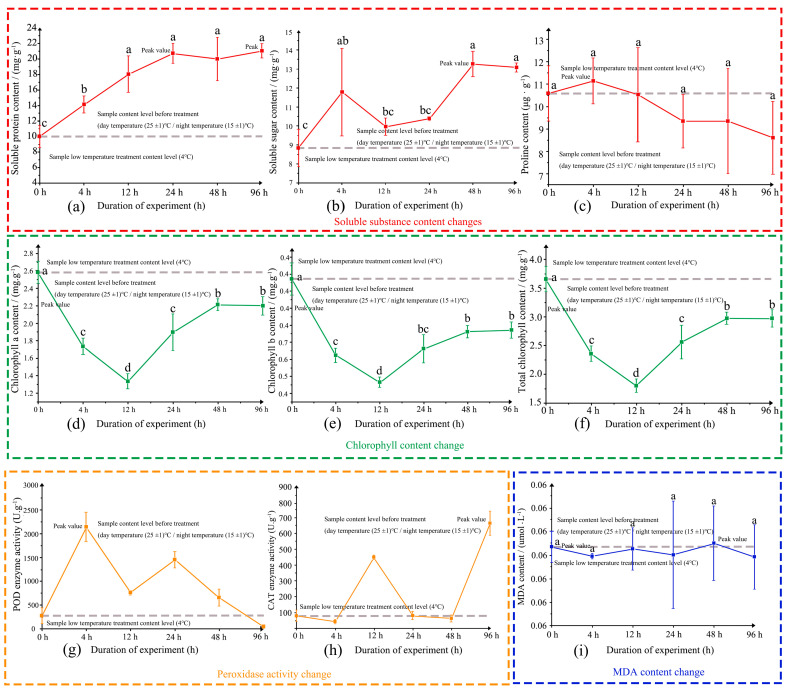
Changes in Physiological Indicators of *C. reticulata* ‘shizitou’ under Low-Temperature Treatment. **Note:** (**a**–**c**) Changes in soluble protein content (**a**), soluble sugar content (**b**), and proline content (**c**) under low-temperature stress (4 °C); (**d**–**f**) Changes in chlorophyll a content (**d**), chlorophyll b content (**e**), and total chlorophyll content (**f**) during low-temperature stress; (**g**,**h**) Variations in peroxidase (POD) activity (**g**) and catalase (CAT) activity (**h**) in response to low-temperature treatment; (**i**) Changes in malondialdehyde (MDA) content under low-temperature stress. a–d: Changes in Physiological Indicators of *C. reticulata* ‘shizitou’ under Low-Temperature Treatment.

The soluble protein content increased progressively with the duration of low-temperature stress, exhibiting a significant rise from 0 h to 24 h (Figure 1a). Between 24 h and 96 h, the soluble protein content continued to increase, although this change was not statistically significant. The lowest content in the control group was 10.0 mg/g, whereas levels at all treatment stages were higher than that of the control. The maximum soluble protein content reached 21.0 mg/g at 96 h, approximately 2.10 times that of the control.

The soluble sugar content displayed a fluctuating yet overall increasing trend under low-temperature stress (Figure 1b). A significant increase was observed from 0 h to 4 h, followed by a non-significant decline from 4 h to 12 h. Subsequently, soluble sugar levels rose significantly from 12 h to 96 h. The minimum content was recorded at 0 h (8.8 mg/g), and the peak content reached 13.2 mg/g at 48 h, approximately 1.50 times higher than that of the control.

Proline content initially increased and then decreased in response to low-temperature stress (Figure 1c). Although an increase was observed from 0 h to 4 h, this change was not statistically significant. From 4 h to 96 h, proline content declined gradually, but again, the change was not significant. The lowest content was 8.5 μg/g at 96 h, while the peak content of 11.1 μg/g at 4 h was about 1.05 times that of the control.

#### 3.1.2. Changes of Chlorophyll Content in Low-Temperature Stress Treatment

With the prolongation of low-temperature stress, the contents of chlorophyll a, chlorophyll b, and total chlorophyll initially decreased and subsequently increased (Figure 1d–f). From 0 h to 12 h, all chlorophyll contents declined significantly, reaching their lowest levels at 12 h. Thereafter, from 12 h to 96 h, the contents increased significantly. The highest chlorophyll content was observed at 0 h (3.6 mg/g), while the lowest was recorded at 12 h (1.7 mg/g).

#### 3.1.3. Changes of Peroxidase Activity During Low-Temperature Stress Treatment

With the extension of low-temperature stress, POD (peroxidase) activity exhibited a fluctuating trend (Figure 1g). POD activity increased significantly from 0 h to 4 h, decreased significantly from 4 h to 12 h, rose again significantly from 12 h to 24 h, and then declined significantly from 24 h to 96 h. The highest POD activity was recorded at 4 h (2140.0 U/g), which was 7.8 times higher than that of the control, while the lowest activity was observed at 96 h (43.8 U/g).

CAT (catalase) activity showed a biphasic response to low-temperature stress (Figure 1h). It initially increased from 0 h to 4 h, followed by a decrease from 4 h to 12 h. Subsequently, CAT activity continued to decline from 12 h to 48 h and then increased again from 48 h to 96 h. The minimum CAT activity was detected at 4 h (41.7 U/g), while the maximum activity was observed at 96 h (661.7 U/g), approximately 8.3 times that of the control.

#### 3.1.4. Change of Malondialdehyde Content in Low-Temperature Stress Treatment

With the extension of low-temperature stress, malondialdehyde (MDA) content exhibited a fluctuating but overall decreasing trend, although the changes were not statistically significant (Figure 1i). The highest MDA content was observed at 48 h (0.09 μmol/g), approximately 1.02 times that of the control, while the lowest content was recorded at 96 h (0.08 μmol/g).

#### 3.1.5. Correlation Analysis of Physiological Indicators

Correlation analysis of various physiological indices under low-temperature treatment (Table 2) revealed that soluble protein content was positively correlated with soluble sugar content. Chlorophyll b content showed a negative correlation with both soluble protein content and POD activity. Additionally, POD activity was negatively correlated with chlorophyll a content, total chlorophyll content, and CAT activity. Chlorophyll a content was positively correlated with both chlorophyll b and total chlorophyll contents, and a significant positive correlation was also observed between chlorophyll b and total chlorophyll contents. No significant correlations were detected among the other indices.

### 3.2. Transcriptome Analysis of C. reticulata ‘Shizhitou’ Leaves Under Cold Stress

#### 3.2.1. Sequencing and Annotation

Transcriptome sequencing of 18 samples was successfully completed, generating a total of 120.95 Gb of clean data. Each sample produced at least 6.20 Gb of clean data, with a Q30 base percentage exceeding 88.92%, indicating high sequencing quality. Following de novo assembly, a total of 56,424 unigenes were obtained, with an N50 length of 2007 bp, reflecting high assembly integrity. Notably, 16,904 unigenes were longer than 1 kb. Functional annotation identified 39,278 unigenes with successful annotations (Supplementary Table S1). Overall, the sequencing and assembly quality were robust, providing a reliable basis for subsequent analyses.

#### 3.2.2. Analysis of Gene Expression Distribution

Gene expression levels (FPKM) exhibited high similarity in expression patterns among samples (Supplementary Figure S1a). The reproducibility of samples within and between groups was strong, with correlation coefficients exceeding 0.98 within each group (Supplementary Figure S1b). Principal component analysis (PCA) of the transcriptome data (Supplementary Figure S1c) revealed that samples from the six low-temperature treatment groups were relatively dispersed, indicating distinct differences between groups, while samples within each group were closely clustered, reflecting high internal consistency. Overall, the gene expression data demonstrated good reproducibility and reliability, supporting subsequent analyses.

**Note:** Figure S1a shows the distribution of sample gene expression. Curves of different colors represent different samples. The horizontal coordinate of points on the curve represents the pair value of FPKM corresponding to the sample, and the vertical coordinate of points represents the probability density. In the Figure S1b sample correlation heat map, different colors in the heat map indicate the strength of the correlation between different samples. The darker the color, the higher the correlation, and the lighter the color, the lower the correlation. In the Figure S1c sample principal component analysis diagram, different coordinates represent different principal components, the percentage represents the contribution value of corresponding principal components to sample differences, each point represents a sample, and samples in different groups are represented by different colors and shapes.

#### 3.2.3. Differential Expression Gene Analysis

**Figure 2:**
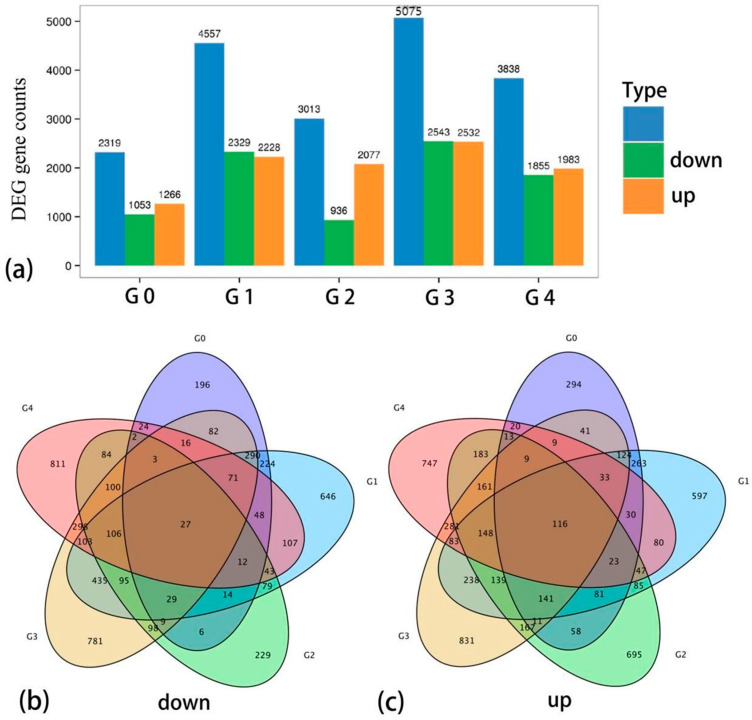
Statistical map of differential genes. **Note:** Horizontal coordinates represent different sets of differential genes, blue represents all differential genes, orange represents up-regulated genes, green represents down-regulated genes, and vertical coordinates represent the number of differential genes. (**a**–**c**) Statistical visualization of differential gene expression.

A total of 10,816 differentially expressed genes were screened with FDR < 0.05 (FDR value was q-value) and |log2FC| > 1 as thresholds. Among them, 5748 genes were significantly up-regulated after low-temperature stress, while 5068 genes were significantly down-regulated (Figure 2a). Six groups were set for comparison between normal temperature control and 4 °C continuous low-temperature treatment at five time points, respectively: G0: CKvsT1; G1: CKvsT2; G2: CKvsT3; G3: CKvsT4; G4: CKvsT5. The results showed that 294 genes were upregulated only in G0, 597 genes were upregulated only in G1, 695 genes were upregulated only in G2, 831 genes were upregulated only in G3, 47 genes were upregulated only in G4, and 116 genes were upregulated jointly in five groups (Figure 2c). There were 196 genes down-regulated only in G0, 646 genes down-regulated only in G1, 229 genes down-regulated only in G2, 781 genes down-regulated only in G3, 811 genes up-regulated only in G4, and 27 genes co-down-regulated in five groups (Figure 2b).

#### 3.2.4. GO Annotation, KEGG Enrichment, and Transcription Factor Analysis of Differentially Expressed Genes

**Figure 3:**
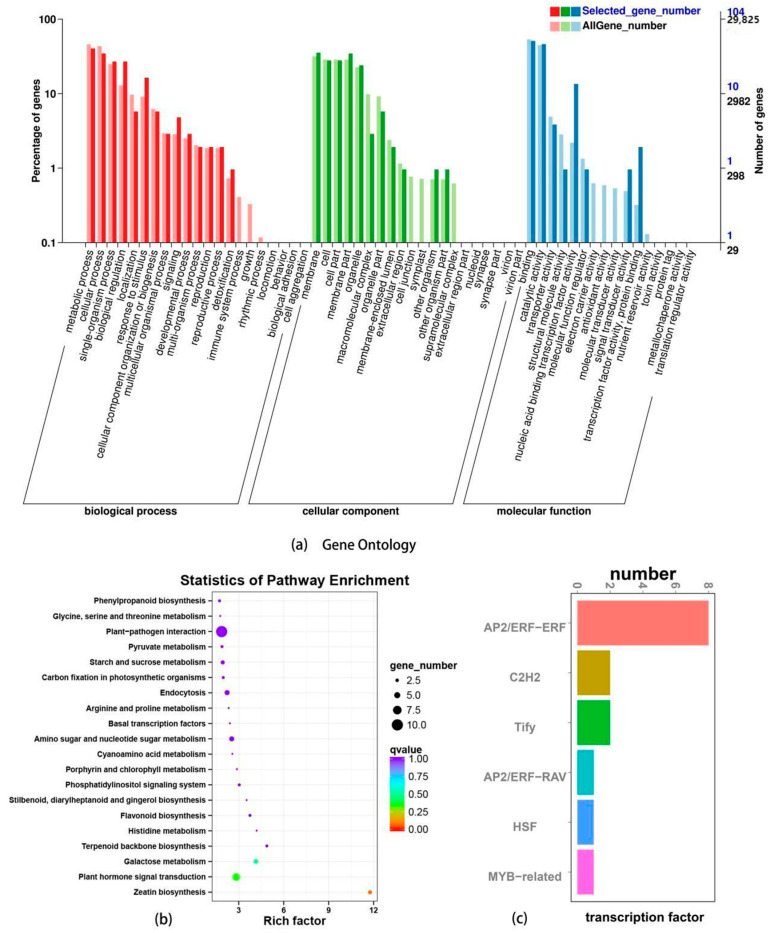
Analysis of codifferential genes in the transcriptome. Note: (**a**) Gene Ontology (GO) enrichment analysis. (**b**) Rich factor analysis of KEGG pathways. (**c**) Transcription factor (TF) binding motif enrichment.

The 143 genes with common differential expression were annotated by GO (Figure 3a). The results showed that in terms of biological processes (BP), the top three notes were metabolic process (GO:0008152) and cellular process (BP), GO:0009987), single-organism process (GO:0044699), and biological regulation (GO:0065007). In terms of cell components (CC), the top three are membrane (GO: 0016020), cell (GO:0005623) and cell part (GO:0044464), and membrane part (GO:0044425). In terms of molecular function (MF), the top three expressions were binding (GO:0005488), catalytic activity (GO:0003824), and transporter activity (GO:0005215).

The 143 genes with common differential expression were analyzed by KEGG pathway enrichment. The 20 most significant KEGG pathways were selected, and scatter plots were drawn (Figure 3b). The results showed that differentially expressed genes were significantly enriched in the Zeatin biosynthesis (ko00908) metabolic pathway under low-temperature stress induction, followed by Plant hormone signal transduction Plant hormone signal transduction, ko04075, Plant hormone Signal transduction, Plant hormone Signal transduction, and Galactose metabolism, ko00052. In the enrichment pathway of Zeatin biosynthesis (ko00908), three genes were located, and the expression of these three genes showed a trend of up-regulation.

Using |log2FC| > 1 as the limit, the transcription factors that may be involved in the regulation were accurately mined. Six gene families with significant correlation were screened, namely, the AP2/ERF transcription factor (including two subfamilies AP2/ERF-ERF and AP2/ERF-RAV), zinc finger protein transcription factor, and zinc finger protein transcription factor (Figure 3c), as well as the TIFY transcription factor, heat shock factor (HSF) transcription factor, MYB transcription factor. A total of 23 transcription factors were identified in six gene families.

#### 3.2.5. Localization Gene Verification

**Figure 4:**
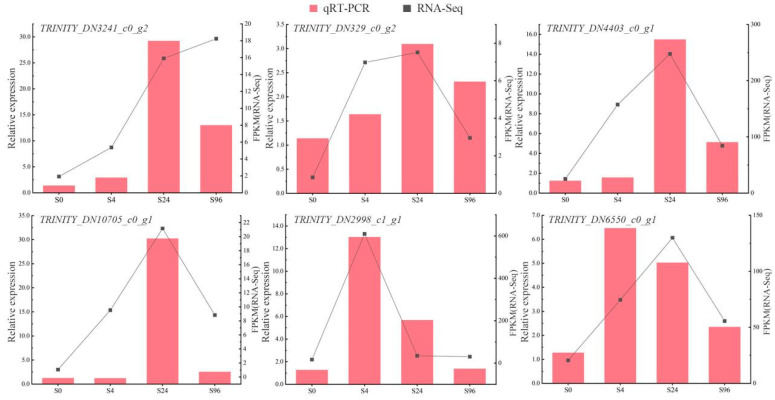
qRT-PCR verification of transcriptome data of response to cold stress.

To validate the accuracy of RNA-Seq data, 10 randomly selected co-expressed genes were subjected to quantitative PCR (qPCR) verification. The results (Figure 4) demonstrated that the expression trends of these genes in RNA-Seq data were highly concordant with those from the transcriptomic analysis. The qRT-PCR results further corroborated the sequencing data, confirming the reliability of the transcriptome sequencing outcomes.

## 4. Discussion

### 4.1. Physiological Responses to Cold Stress in C. reticulata

Our results demonstrate that prolonged cold stress induced a sustained increase in soluble protein content, consistent with findings in *C. sinensis*, where CsLEA2 (Late Embryogenesis Abundant protein) and CsCOR413 (Cold-Regulated protein) enhanced dehydration tolerance [31]. However, soluble sugars exhibited a fluctuating trend, similar to *C. sinensis*, where CsSPS (sucrose-phosphate synthase) showed transient upregulation followed by downregulation, likely due to metabolic reallocation [32]. In our study, soluble sugar levels peaked at 24 h but declined by 48 h, suggesting an initial osmotic adjustment phase followed by energy redirection towards long-term cold adaptation.

The recovery of chlorophyll content after initial degradation aligns with *C. sinensis*, where CsCHL (chlorophyll synthase) restored chlorophyll biosynthesis under sustained cold exposure [33]. However, in our study, the recovery was slower than in *C. sinensis*, possibly due to genetic differences in cold responsiveness among *Camellia* species.

Antioxidant enzyme activities (POD, CAT) displayed an early induction followed by gradual suppression, similar to *C. sinensis*, where CsAPX1 (ascorbate peroxidase) and CsCAT3 (catalase) were initially upregulated but later repressed due to oxidative system fatigue [34]. Notably, we observed a negative correlation between chlorophyll retention and POD activity, supporting previous findings in *C. oleifera*, where excessive CoPOD1 activity contributed to chlorophyll breakdown under prolonged cold [35]. This suggests that overproduction of ROS-scavenging enzymes may disrupt chloroplast stability at later stress stages.

### 4.2. Transcriptomic Mechanisms Underlying Cold Stress Responses

GO enrichment analysis demonstrated a strong bias towards metabolic processes, particularly carbohydrate metabolism, corroborating *C. sinensis* studies where CsBAM1 (β-amylase) and CsSUS2 (sucrose synthase) played key roles in osmoregulation [33]. Our KEGG analysis highlighted zeatin biosynthesis as a key cold-response pathway, with CrLOG2 (cytokinin-activating enzyme) potentially enhancing cold tolerance, as seen in *C. sinensis* [36] Additionally, CrARR12 (response regulator) may mediate cytokinin-ABA crosstalk, similar to CsRR9, which regulates stress-responsive genes in *C. sinensis* [37].

The upregulation of galactose metabolism-related genes (e.g., CrPME41) suggests cell wall remodeling to prevent cold-induced membrane damage, analogous to findings in *C. japonica* [38]. We also identified key transcription factors (TFs), including CrDREB1A (similar to CsDREB1A), which likely regulates cold-responsive genes [39], and CrMYB44, which may enhance proline accumulation [40]. Furthermore, CrHSFA2, previously linked to heat shock protein stabilization in *C. sinensis* [41], was upregulated, suggesting a conserved stress-protective mechanism.

### 4.3. Physiology-Transcriptome Integration: Unifying Mechanisms

Our study reveals a biphasic pattern of SOD/POD activity, characterized by an initial surge at 4 h, decline at 24 h, and subsequent rebound at 48 h, which cannot be fully explained by transcriptional changes alone. This suggests multi-layered post-transcriptional regulation of antioxidant enzymes. The early activity peak (4 h) likely reflects ROS-induced post-translational modifications [42] rather than immediate gene induction. The subsequent activity decline (24 h) occurs despite sustained high expression of genes like APX1, possibly due to feedback inhibition or targeted protein degradation. Intriguingly, the late-stage rebound (48 h) coincides with upregulation of zeatin biosynthesis genes, suggesting cytokinin-mediated enzyme stabilization [43] and metabolic shifts toward glutathione cycling, as indicated by KEGG enrichment (glutathione metabolism, ko00480). The initial co-accumulation of soluble sugars and proteins at 24 h aligns with their shared transcriptional regulation, including CrDREB1A, which simultaneously activates genes for both osmotic protectants and antioxidant defense pathways [44]. However, their decoupling at 48 h—marked by declining sugars but persistent protein levels—suggests a strategic shift in resource allocation toward long-term repair mechanisms. This shift is further supported by sustained high expression of membrane stabilization genes (PME41, FAD7) alongside reduced ROS-scavenging demand as chlorophyll levels gradually recover [44].

## 5. Conclusions

This study demonstrates that *C. reticulata* employs a multi-dimensional adaptation strategy in response to low-temperature stress, integrating physiological regulation, metabolic reprogramming, and transcriptional control. The accumulation of osmotic protectants (soluble sugars, proteins) and the dynamic regulation of antioxidant enzymes (POD, CAT, SOD) effectively mitigate oxidative damage, maintaining membrane stability and photosynthetic function. Transcriptome analysis reveals key metabolic pathways (galactose metabolism, zeatin synthesis) and transcription factor networks (AP2/ERF, MYB, DREB/CBF) that orchestrate cold adaptation by modulating hormone signaling (zeatin, ABA) and ROS scavenging.

While this work uncovers the molecular–physiological basis of cold resistance, further research is needed to bridge these findings with breeding applications. Specifically, functional validation of candidate genes (e.g., DREB/CBF, HSF) and metabolic markers could enable precise genetic improvement, enhancing cold tolerance in *C. reticulata* cultivars. By linking mechanistic insights to trait selection and gene-editing strategies, this study lays a theoretical foundation for developing climate-resilient germplasm.

## Figures and Tables

**Table 1:** Genes and primers used for qRT-PCR.

Gene Name	Gene ID	Primer Sequence (5′→3′)
*HSF*	TRINITY_DN3241_c0_g2	F: CGCTTGAAGCTTTACAGGGC
		R: TTCCTCGGAAGTACGAGCCT
*CCT*	TRINITY_DN329_c0_g2	F: CGGACATCCATAAGGCGACA
		R: CGTTTTCGATGTTCCGAGGC
*TIFY*	TRINITY_DN4403_c0_g1	F: AACTCCGGTGACGAGAAACC
		R: CTTTGCCTTATCAGCCGGGA
*AP2*	TRINITY_DN10705_c0_g1	F: AGGGGTCGTAGGCTATGGTT
		R: TGGCTCGAAGTTGTTGTGGA
*C2H2*	TRINITY_DN2998_c1_g1	F:GGCAAACTCTAGCCCGCATA
		R: GAGACCACATCGACCAAGGG
*AP2*	TRINITY_DN6550_c0_g1	F: ACGTCTTTTCCGGCGATTCT
		R: GAGGATTTGGCTCGGGACTT

**Table 2:** Correlation analysis of physiological indexes of *C. reticulata* ‘shizhitou’ low-temperature stress.

	Soluble Protein	Soluble Sugar	MDA	Chlorophyll a	Chlorophyll b	Total Chlorophyll	Proline	POD	CAT
Soluble protein	1								
Soluble sugar	0.493 *	1							
MDA	−0.131	−0.108	1						
chlorophyll a	−0.269	0.041	0.096	1					
chlorophyll b	−0.473 *	−0.146	0.07	0.963 **	1				
total chlorophyll	−0.336	−0.018	0.088	0.996 **	0.983 **	1			
proline	−0.445	−0.373	0.132	−0.193	−0.079	−0.158	1		
POD	−0.072	−0.005	−0.118	−0.512 *	−0.486 *	−0.508 *	0.283	1	
CAT	0.404	0.215	−0.068	−0.199	−0.24	−0.214	−0.291	−0.555 *	1

** *p*  < 0.01 and * *p*  < 0.05. Extremely significant difference: *p* < 0.01 **; significant difference: *p* < 0.05 *.

## Data Availability

The original contributions presented in the study are included in the article/Supplementary Material, further inquiries can be directed to the corresponding author.

## Supplementary Materials

The following supporting information can be downloaded at: https://www.mdpi.com/article/10.3390/genes16050503/s1, Figure S1: Analysis of distribution of gene expression in samples; Table S1: Unigene notes statistical table.
