# Supplementary material for: Physiological Responses and Transcriptome Analysis of Camellia reticulata Under Low-Temperature Stress
Source: Genes (Basel). 2025 Apr 27;16(5):503. doi: 10.3390/genes16050503 (PMC12110766; doi:10.3390/genes16050503)
Supplement: Supplementary file 1 [file genes-16-00503-s001.zip › genes-3567780-supplementary.pdf]

Supplementary Figure and Table

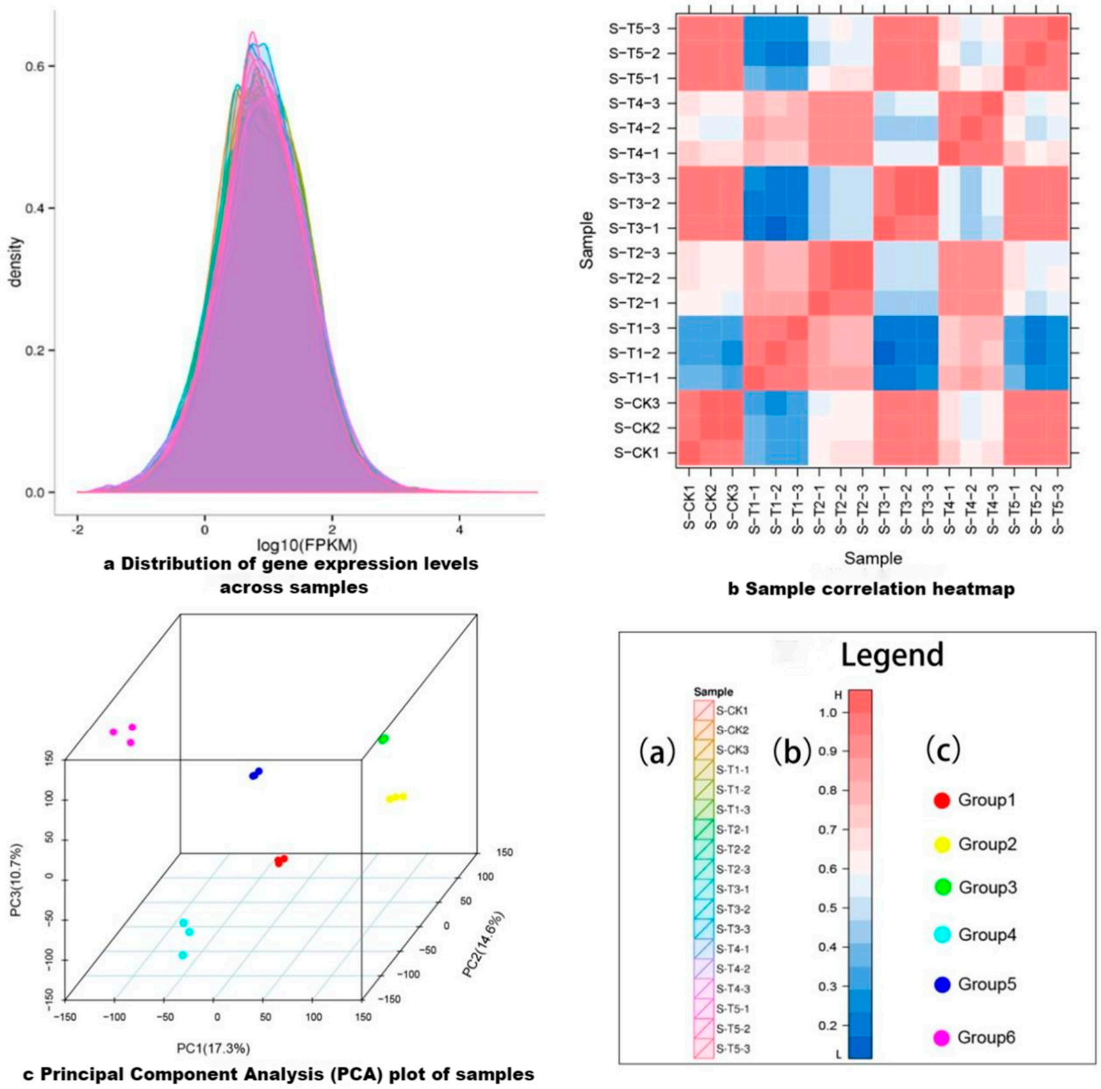

Figure S1. Analysis of distribution of gene expression in samples.

Table S1. Unigene notes statistical table.

#Anno_Databas	Annotated_Number	300<=length<1000	length>=1000
COG_Annotation	7909	2781	5128
GO_Annotation	29825	16196	13622
KEGG_Annotation	22256	10776	11480
KOG_Annotation	18179	8645	9534
Pfam_Annotation	21624	8853	12771
Swissprot_Annotation	21914	10204	11710
TrEMBL_Annotation	36588	20517	16071
eggNOG_Annotation	27848	14091	13757
nr_Annotation	38657	22424	16233
